# The Good, the Bad, and the Epigenetic: Stress-Induced Metabolite Regulation and Transgenerational Effects

**DOI:** 10.3390/epigenomes9020010

**Published:** 2025-03-29

**Authors:** Saida Ibragić, Sabina Dahija, Erna Karalija

**Affiliations:** 1 Department of Chemistry, Faculty of Science, University of Sarajevo, Zmaja od Bosne 33-35, 71000 Sarajevo, Bosnia and Herzegovina; saida.i@pmd.unsa.ba; 2 Laboratory for Plant Physiology, Department of Biology, Faculty of Science, University of Sarajevo, Zmaja od Bosne 33-35, 71000 Sarajevo, Bosnia and Herzegovina; sabina.dahija@pmf.unsa.ba

**Keywords:** epigenetic regulation, stress memory, secondary metabolites, phenylpropanoids, DNA methylation, histone modifications, non-coding RNAs

## Abstract

Background: Plants face a wide range of environmental stresses that disrupt growth and productivity. To survive and adapt, they undergo complex metabolic reprogramming by redirecting carbon and nitrogen fluxes toward the biosynthesis of protective secondary metabolites such as phenylpropanoids, flavonoids, and lignin. Recent research has revealed that these stress-induced metabolic processes are tightly regulated by epigenetic mechanisms, including DNA methylation, histone modifications, chromatin remodeling, and non-coding RNAs. Methods: This review synthesizes current findings from studies on both model and crop plants, examining the roles of key epigenetic regulators in controlling secondary metabolism under stress. Special focus is placed on dynamic changes in DNA methylation, histone acetylation, and the action of small RNAs such as siRNAs and miRNAs in transcriptional and post-transcriptional regulation. Results: Evidence indicates that stress triggers rapid and reversible epigenetic modifications that modulate gene expression linked to secondary metabolic pathways. These modifications not only facilitate immediate metabolic responses but can also contribute to stress memory. In some cases, this memory is retained and transmitted to the next generation, influencing progeny stress responses. However, critical knowledge gaps remain, particularly concerning the temporal dynamics, tissue specificity, and long-term stability of these epigenetic marks in crops. Conclusions: Understanding how epigenetic regulation governs secondary metabolite production offers promising avenues to enhance crop resilience and productivity in the context of climate change. Future research should prioritize dissecting the stability and heritability of these modifications to support the development of epigenetically informed breeding strategies.

## 1. Introduction

Plants are frequently exposed to a variety of environmental and/or biotic stresses and thus are equipped with defensive cellular, molecular, and biochemical mechanisms to protect themselves and counteract unfavorable conditions for their growth and development [1,2]. In response to environmental stimuli, which can lead to morphological changes, reductions in physiological and yield responses, early senescence, and even cell death [3], plants synthesize secondary metabolites (SMs) that are comprised of 200,000 isolated and characterized compounds so far [4], including over 8000 phenylpropanoids, 12,000 alkaloids, and 40,000 terpenoids [5]. Stress-exposed plants are susceptible to epigenetic modifications that result in adaptive transgenerational plasticity, where their offspring exhibit morphological changes in the form of increased trichome density and leaf number that enhance stress resistance, potentially affecting the production of secondary metabolites involved in defense and adaptation.

Despite considerable progress in understanding epigenetic regulation of plant metabolic pathways under stress, several key research gaps remain. One of the most prominent gaps is the incomplete understanding of small RNA (sRNA)-guided epigenetic inheritance. While studies have demonstrated that sRNAs, including siRNAs and miRNAs, participate in the establishment of stress-induced DNA methylation marks and chromatin modifications, the mechanisms governing their long-distance mobility and their ability to direct heritable epigenetic changes across generations are not yet fully understood [6]. In particular, the molecular specificity by which certain sRNAs target genes associated with secondary metabolism and stress adaptation in reproductive tissues remains unclear, which could potentially be used in crop breeding for transgenerational resilience [7].

Another challenge lies in the absence of comprehensive, genome-wide temporal maps of histone modifications during dynamic stress cycles and recovery phases. While static profiles of histone acetylation and methylation are available for some model plants, the lack of high-resolution time-series datasets hinders understanding of how chromatin landscapes evolve during prolonged or recurring stress [8,9]. Additionally, although advances in CRISPR/dCas9-based epigenome editing tools have been made, their application in modulating specific metabolic pathways remains limited due to concerns regarding off-target effects and epigenetic mark stability over multiple generations [10]. Addressing these gaps will require the integration of multi-omics technologies, advanced imaging of chromatin dynamics, and the development of precise epigenetic editing platforms tailored to complex plant genomes.

This review aims to examine how epigenetic modifications control plant secondary metabolite production during stress, highlight key molecular players, and explore their potential for crop improvement. We hypothesize that integrated epigenetic mechanisms reinforce both transient and transgenerational metabolite memory, enabling sustainable agricultural resilience.

## 2. Secondary Metabolites—Origin and Synthesis Roles in Plants

**Figure 1:**
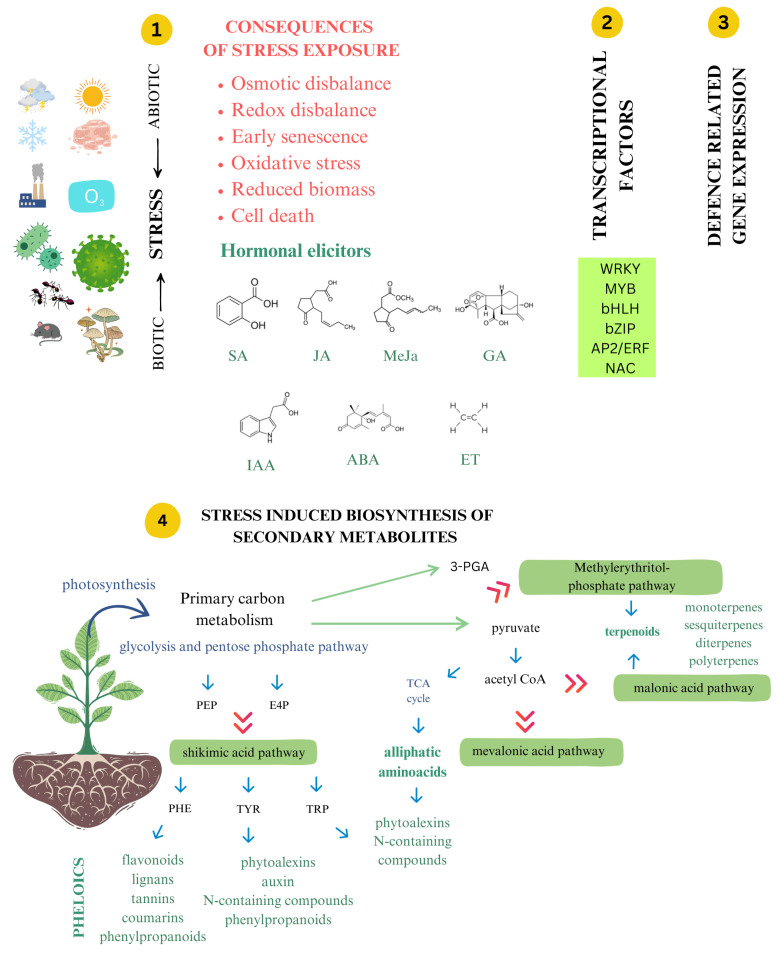
Biosynthesis pathways of main SMs in plants under stress exposure.

Being structurally diverse, ranging from simple rings to highly complex polymers, SMs act as signaling molecules, protecting plants from herbivores, mitigating oxidative damage, orchestrating adaptive responses, and enabling ecological relationships between plants and other organisms. Most commonly, based on their chemical structure, SMs are classified into three major groups: phenolics (phenolic acids, flavonoids, stilbenes, lignins, tannins), terpenes (isoterpenoids, terpenoids), and *n*-containing compounds (alkaloids, glycosides, glycosinates) [11]. Depending on their volatility, SMs can be classified as volatile or non-volatile compounds. Different SMs accumulate selectively in various plant parts based on specific stress conditions. This process is fine-tuned and regulated at the molecular level by genes and transcription factors (Figure 1). While the metabolic pathways producing these molecules have been extensively studied, the role of epigenetic regulation in modulating their synthesis remains underexplored. Plants’ SMs are derived from three distinct biosynthetic pathways. The shikimate pathway leads to the production of aromatic amino acids that are building blocks of proteins and precursors of phenolics and *n*-containing compounds. The tricarboxylic acid cycle pathway is also responsible for the synthesis of *n*-containing compounds, while the mevalonic pathway generates terpenes [12,13]. The biosynthesis of secondary metabolites requires significant energy due to complex, resource-intensive pathways. Since they are not essential for basic cellular functions, their production is less prioritized. Energy investment varies based on the compound and environmental cues, requiring a balance between sustaining life and enhancing survival [14]. Phenolic compounds, as widely distributed SMs in plants, act as potent antioxidants by donating electrons to neutralize reactive oxygen species (ROS), with products (phenoxy radicals) being less reactive than ROS, preventing further oxidative stress. Orthodihydroxy phenolics are more effective ROS scavengers than monohydroxy phenolics, while flavonoids gain enhanced antioxidant activity from a C ring double bond and hydroxyl groups [15]. Lignin, furanocoumarins, and tannins are good examples to illustrate the protective roles of phenolics. Lignin supports plant growth, structural integrity, and stress tolerance by strengthening tissues and deterring herbivores. Furanocoumarins influence insect feeding and development while sensitizing cells to light. Isoflavonoids aid in phytoalexin production during plant–microbe interactions. Tannins, classified as condensed or hydrolysable, deter herbivores and enhance disease resistance. Their accumulation is influenced by environmental factors like CO_2_ levels, temperature, light, and nutrient availability [16,17,18]. Terpenes, formed from the derivatives of glycolytic or acetyl CoA intermediates, mostly act as defensive toxins and herbivore deterrents. Alkaloids, cyanogenic glysocides, glucosinolates, and non-protein amino acids as *n*-containing compounds can also be synthesized in response to both biotic and abiotic stresses.

Biotic and abiotic stresses intensify the synthesis of SM production (with examples). SA—salicylic acid, JA—jasmonic acid, MeJa—methyl jasmonate, GA—gibberellic acid, IAA—indole-3-acetic acid, ABA—abscisic acid, ET—ethylene, PEP—phosphoenol pyruvate, E4P—erythrose 4-phosphate, PHE—phenylalanine, TYR—tyrosine, TRP—tryptophan, 3-PGA—3-phosphoglycerate.

There are numerous examples of how plant SMs play a pivotal role in mitigating a wide range of environmental stresses such as temperature, drought, salinity, and UV irradiation. Salinity stress causes cellular dehydration, disrupting osmotic balance and triggering SM accumulation to mitigate oxidative damage. Plants adapt by synthesizing essential SMs, maintaining membrane stability, nutrient balance, and redox homeostasis despite salt-induced water scarcity [19]. Under salinity stress, pepper plants (*Capsicum annuum*) increase hydroxycinnamic acids, capsaicinoids, phenols, and flavonoids, enhancing stress tolerance [20]. Drought stress induces changes in plants, which negatively impact biomass quality and quantity. As reported for sage (*Salvia dolomitica*), drought stress upregulates terpenoid biosynthesis genes, including geranyl diphosphate synthase (GPPS), farnesyl diphosphate synthase (FPPS), geranylgeranyl diphosphate synthase (GGPPS), and copalyl diphosphate synthase 2 (CPS2) [21].

Light quality also affects the synthesis of bioactive compounds and SMs in plants. Key light factors include photoperiod, intensity, and quality. Different plant species respond differently to light variations, adapting by regulating SM accumulation and release. Phenolic compounds, triterpenoids, and flavonoids, valued for their antioxidant properties, are among the SMs heavily influenced by light exposure [22,23].

Stress induced by heavy metal exposure disrupts plant metabolic activity, affecting sugar production, photosynthetic pigments, and protein synthesis by inhibiting key enzymes. It also alters secondary metabolism, influencing the synthesis of bioactive compounds [24]. For example, artemisinin, a sesquiterpene compound, is induced in sweet sagewort (*Artemisia annua*) when exposed to As toxicity [25]. Similarly, lignin, a phenolic compound, forms a protective barrier in the cytoderm of cotton, preventing Cd absorption through root tissues [26]. Acute exposure to high O_3_ concentrations causes necrosis and chlorophyll loss in sensitive plants, while chronic exposure to lower levels accelerates leaf senescence [27]. In response to elevated O_3_, plants show changes in phenols, flavonoids, and phenolic acids (e.g., caffeic and rosmarinic acids), playing a key role in antioxidant defense and detoxification [28].

## 3. Epigenetic Modifications in Plants Related to Metabolite Production

Epigenetic modifications in plants are central regulatory mechanisms that influence gene expression without altering the underlying DNA sequence. They play vital roles in growth, development, stress response, and metabolic adjustments. These modifications include DNA methylation, histone modifications, chromatin remodeling, and the action of non-coding RNAs (ncRNAs), all of which interact in a highly coordinated manner.

### 3.1. Epigenetic Modifications at the DNA Level

Epigenetic modifications at the DNA level are the most extensively studied epigenetic modification in plants, evident in cytosine methylation, which occur in three sequence contexts: CG, CHG, and CHH (where H is A, T, or C). This modification is catalyzed by specific DNA methyltransferases, including METHYLTRANSFERASE 1 (MET1) for maintenance methylation in the CG context, CHROMOMETHYLASE 3 (CMT3) for CHG methylation, and DOMAINS REARRANGED METHYLTRANSFERASE 2 (DRM2), which mediates de novo methylation via the RNA-directed DNA methylation (RdDM) pathway [29]. These methylation patterns are not static but are dynamically reconfigured in response to environmental cues, allowing plants to fine-tune the expression of genes associated with growth, stress responses, and metabolic adjustments.

Stress-induced DNA methylation changes can silence or activate genes involved in secondary metabolite biosynthesis pathways, such as the phenylpropanoid, terpenoid, and alkaloid pathways. For example, drought stress in *Arabidopsis thaliana* results in promoter demethylation of proline biosynthesis genes, contributing to osmotic adjustment and long-term stress priming. Similarly, under salinity stress, *Capsicum annuum* exhibits altered DNA methylation in genes regulating capsaicinoid and flavonoid biosynthesis, enhancing plant resilience and antioxidant capacity [30].

DNA demethylation, catalyzed by enzymes such as REPRESSOR OF SILENCING 1 (ROS1) and DEMETER (DME), also plays an essential role in reprogramming metabolic responses. These demethylases enable the activation of stress-responsive genes, including those involved in antioxidant and lignin biosynthesis [31]. Notably, such methylation adjustments are sometimes retained as part of stress memory, allowing progeny to inherit primed states. In *Arabidopsis*, certain methylation patterns established under drought stress could persist across generations, enhancing the plant’s response to subsequent stress events [32].

### 3.2. Histone Modifications

**Figure 2:**
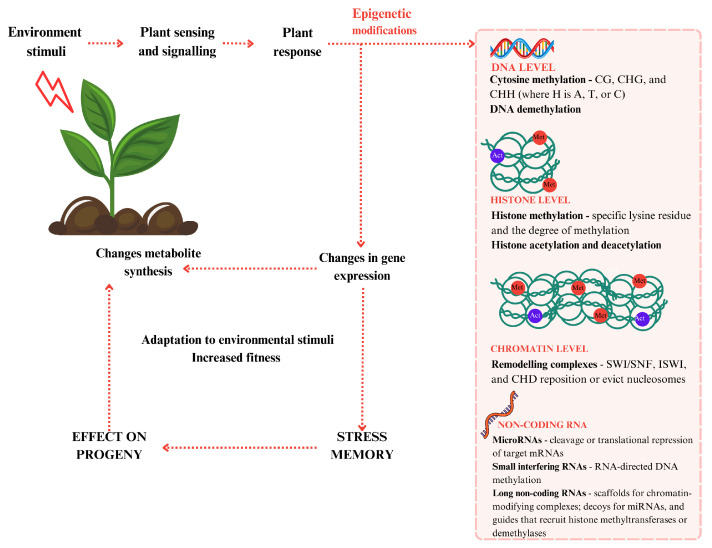
Epigenetic mechanisms in regulation of gene expression and its effects on stress memory.

Epigenetic regulation at the histone level involves covalent post-translational modifications of histone proteins, including acetylation, methylation, phosphorylation, ubiquitination, and sumoylation (Figure 2). These modifications alter chromatin structure, influencing the accessibility of transcriptional machinery to DNA and fine-tuning gene expression in response to environmental stress. Among these, histone acetylation and methylation are the most extensively studied in relation to stress-induced secondary metabolite biosynthesis.

Histone acetylation, catalyzed by histone acetyltransferases (HATs), generally correlates with transcriptional activation by loosening chromatin and allowing for transcription factor binding. In contrast, histone deacetylation by histone deacetylases (HDACs) compacts chromatin and represses gene expression. Stress conditions have been shown to increase acetylation at key metabolic gene promoters. For example, in *Arabidopsis thaliana*, drought stress leads to hyperacetylation of histones H3 and H4 at the promoters of phenylpropanoid biosynthetic genes (PAL, CHS, and F3H), facilitating rapid induction of flavonoid biosynthesis [33]. Similarly, in rice, histone acetylation changes have been linked to enhanced water-use efficiency and stress resilience [34].

Histone methylation exhibits a more complex regulatory function. Depending on the specific lysine residue and the degree of methylation (mono-, di-, or tri-methylation), it can either activate or repress transcription. Active marks such as H3K4me3 and H3K36me3 promote gene expression, while repressive marks like H3K27me3 and H3K9me2 are associated with gene silencing. Stress-induced reprogramming of these epigenetic marks modulates secondary metabolism genes. For instance, cold stress has been reported to reduce H3K27me3 at anthocyanin biosynthesis gene loci, leading to increased pigment accumulation as part of the antioxidant defense system [35].

### 3.3. Chromatin Remodeling

In addition to histone modifications, chromatin remodeling complexes such as SWI/SNF, ISWI, and CHD families are also regulators of chromatin accessibility. These complexes use ATP hydrolysis to reposition or eject nucleosomes, enabling rapid activation or repression of stress-responsive genes. The SWI/SNF complex, particularly components like BRAHMA (BRM), has been shown to interact with transcription factors (WRKY and MYB) to modulate secondary metabolite pathway genes in response to pathogen attack and abiotic stress [36]. Moreover, chromatin remodeling is essential for balancing growth–defense trade-offs, ensuring that metabolic investment in secondary metabolite production occurs without compromising plant development (Figure 2).

Recent studies also indicate that stress memory can be maintained at the histone level, where stress-induced modifications, such as persistent H3K4me3 and partial retention of bivalent chromatin marks (H3K4me3/H3K27me3), keep key metabolic genes in a ‘poised’ state for faster reactivation during recurring stress cycles [37].

### 3.4. Non-Coding RNAs

Non-coding RNAs (ncRNAs), including microRNAs (miRNAs), small interfering RNAs (siRNAs), and long non-coding RNAs (lncRNAs), have emerged as central regulators of plant gene expression, contributing to the dynamic modulation of growth, development, and stress responses through epigenetic mechanisms (Figure 2). These RNAs modulate chromatin states, DNA methylation, and histone modifications, thereby influencing the expression of key genes involved in metabolism and environmental response [38].

MicroRNAs (miRNAs) are 20–24 nucleotide-long molecules that mediate post-transcriptional gene silencing by guiding the cleavage or translational repression of target mRNAs. Stress-induced miRNAs, such as miR156, miR172, and miR319, target transcription factors that regulate secondary metabolism and stress tolerance pathways [29]. For example, miR858 modulates the phenylpropanoid pathway by targeting MYB transcription factors, directly influencing lignin and flavonoid biosynthesis [39]. miRNAs can also indirectly shape epigenetic landscapes by affecting the abundance of chromatin remodelers and DNA methyltransferases [40].

Small interfering RNAs (siRNAs) are key components of RNA-directed DNA methylation (RdDM), a pathway that leads to transcriptional silencing of transposable elements and stress-responsive genes via DNA methylation and histone modifications. The Dicer-like proteins (DCL3), Argonaute proteins (AGO4), and RNA-dependent RNA polymerase (RDR2) coordinate the biogenesis and function of siRNAs, which guide the DNA methylation machinery to specific loci under stress conditions [41].

Long non-coding RNAs (lncRNAs) (>200 nucleotides) play more diverse roles, acting as scaffolds for chromatin-modifying complexes, decoys for miRNAs, and guides that recruit histone methyltransferases or demethylases to target loci. For instance, the Arabidopsis lncRNA *COLDAIR* mediates vernalization-induced epigenetic silencing of the *FLC* locus by recruiting Polycomb Repressive Complex 2 (PRC2) [42]. Under drought and salinity stress, lncRNAs have been shown to regulate secondary metabolism and stress tolerance by modulating transcription factors and key biosynthetic genes [43].

Moreover, ncRNAs are increasingly recognized as systemic signaling molecules. Mobile siRNAs and miRNAs have been detected in phloem sap, suggesting their role in coordinating systemic stress responses [28]. Some stress-induced ncRNAs contribute to priming and stress memory by establishing epigenetic marks that persist across cell divisions or even generations [37].

Collectively, the growing body of evidence positions ncRNAs as crucial players in epigenetic regulation, linking environmental cues with stable chromatin and transcriptional changes that underlie metabolic plasticity and resilience.

## 4. Stress Signaling and Metabolic Reprograming

Plants integrate diverse external signals through sophisticated perception systems, triggering cascades that consequently reprogram metabolism. Signaling begins with membrane-localized receptors such as pattern recognition receptors (PRRs), mechanosensitive ion channels, and receptor-like kinases (RLKs), which detect abiotic cues (osmotic changes, temperature fluctuations) and biotic elicitors (pathogen- or herbivore-associated molecular patterns). Their activation leads to rapid calcium influx and phosphorylation cascades via receptor-like cytoplasmic kinases (RLCKs) and mitogen-activated protein kinases (MAPKs), which are essential for signal transduction [44].

Mechanical stimuli and osmotic stress activate mechanosensitive channels (e.g., MSL family proteins), leading to transient ion fluxes and membrane potential changes that integrate with ROS waves and hormonal signals, fine-tuning metabolic responses [45]. These early signals also trigger retrograde signaling from chloroplasts and mitochondria, which adjust primary carbon fixation and respiratory pathways to optimize precursor availability for secondary metabolite biosynthesis [46].

An often-underestimated layer of signaling involves sugar-sensing networks, where trehalose-6-phosphate (T6P), hexokinase, and SnRK1 kinases act as metabolic checkpoints, coordinating energy status with stress responses and metabolic allocation. These sensors dynamically regulate not only primary pathways but also transcription of key enzymes in the shikimate, phenylpropanoid, and terpenoid pathways under resource-limited conditions [47].

Epigenetic modifications often influence signaling outcomes. Chromatin remodelers (e.g., SWI/SNF complexes) and histone modifiers respond to signal cascades by altering the accessibility of regulator genes such as WRKYs and MYBs, enabling transient and heritable tuning of metabolic reprogramming [36]. Furthermore, stress-induced small RNAs and long non-coding RNAs (lncRNAs) have been identified as key messengers that link early stress signaling with metabolic reconfiguration, either by targeting transcription factors or chromatin regulators [38].

Stress-induced metabolic reprogramming involves not only upregulation of protective metabolites but also active downregulation of resource-intensive pathways. This metabolic rebalancing is facilitated by negative regulators, such as JAZ repressors in the jasmonate pathway and DELLA proteins, which integrate hormonal cross-talk and ensure energy efficiency [48].

### Primary and Secondary Metabolism: Pathways and Crosstalk

Primary metabolism provides structural components and an energy source for plant survival, including photosynthesis, glycolysis, the tricarboxylic acid (TCA) cycle, and nitrogen assimilation. Secondary metabolism, on the other hand, produces specialized compounds such as phenolics, terpenoids, and alkaloids that protect plants from environmental challenges and contribute to ecological interactions [49]. The balance between these metabolic pathways is tightly regulated, and their crosstalk is dynamically adjusted in response to stress signals.

Photosynthetically fixed carbon is directed toward glycolysis and the pentose phosphate pathway (PPP), generating phosphoenolpyruvate (PEP) and erythrose-4-phosphate (E4P), the initial substrates for the shikimate pathway. This pathway leads to the production of aromatic amino acids (phenylalanine, tyrosine, and tryptophan), which are precursors for phenylpropanoids and alkaloids [13]. Under stress conditions, plants redirect carbon flux from primary growth pathways toward secondary metabolite biosynthesis to enhance defense, often leading to growth retardation as a trade-off [50].

Recent studies have shown that enzymes central to primary metabolism, such as phosphoenolpyruvate carboxylase (PEPC) and malate dehydrogenase (MDH), are transcriptionally upregulated during stress to provide additional metabolic intermediates for secondary metabolite production. Furthermore, feedback from secondary metabolite accumulation (such as flavonoids) can modulate photosynthetic electron transport and ROS signaling, highlighting reciprocal regulation [51].

Sugar signaling plays a crucial role in coordinating primary–secondary metabolic flux. Trehalose-6-phosphate (T6P) has been identified as a key regulator that influences the expression of phenylpropanoid and lignin biosynthesis genes under drought and osmotic stress [47]. Moreover, SNF1-related kinases (SnRK1) integrate energy status with metabolic reprogramming by modulating secondary metabolism genes when carbon availability is limited [52].

An emerging aspect of crosstalk involves plastid-to-nucleus retrograde signaling, where metabolites such as methylerythritol cyclodiphosphate (MEcPP), an intermediate of the MEP pathway, influence nuclear gene expression and regulate secondary metabolite pathways, linking plastid metabolism to stress-responsive phenylpropanoid production [53].

Transcription factors also play a key role in primary–secondary metabolism crosstalk. Transcription factors like bZIP and MYB often co-regulate genes involved in both carbon metabolism and specialized metabolite pathways, enabling rapid adjustment of metabolic priorities [54]. Disruptions in this fine balance can impair stress tolerance or reduce energy efficiency, underscoring the evolutionary refinement of these interconnected metabolic networks.

## 5. Plant Memory: Mechanisms and Implications

Plant memory refers to the capability of plants to “remember” past environmental stimuli and respond more efficiently to subsequent exposures. This memory is mediated by molecular and cellular changes, including epigenetic modifications, that regulate gene expression and metabolic pathways. In the context of secondary metabolite production, plant memory enables rapid and enhanced synthesis of bioactive compounds crucial for survival under recurring stresses under conditions of re-occurring stress or in the next generation (progeny).

### 5.1. Concept of Plant Memory

Plant memory refers to the retention of stress signals via epigenetic modifications, allowing plants to “remember” past exposures and respond more effectively to subsequent stresses. This memory is mediated by persistent chromatin marks, such as histone acetylation and DNA methylation [55,56].

Transcriptional memory refers to the ability of plants to retain stress-induced transcriptional states, enabling a faster and more robust response to recurring stress. This memory is mediated by TFs and chromatin modifications that leave stress-response genes in an inactive (suppressed) or active state. For instance, in *Arabidopsis*, dehydration stress primes ABA-responsive genes by maintaining the binding of transcription factors such as ABF and DREB to their target promoters. This priming ensures rapid reactivation of these genes during subsequent dehydration events, facilitating faster adaptation [56].

In the context of secondary metabolites, transcriptional memory ensures the rapid activation of biosynthetic pathways for protective compounds. For example, the priming of transcription factors involved in phenylpropanoid biosynthesis accelerates flavonoid production during recurring UV exposure. This mechanism not only conserves resources during periods of no stress but also enhances resilience when stress conditions recur.

Based on the duration of “memorized” epigenetic modification (that affect plants response to stimuli), plant memory can be categorized into short-term, long-term, and transgenerational memory. The retention of stress imprints can significantly influence secondary metabolite biosynthesis, which plays a crucial role in plant defense, signaling, and adaptation to environmental challenges [57,58].

Short-term memory is maintained from a few hours to days, enabling plants to rapidly respond with their metabolism and gene expression to external stimuli. Long-term memory, on the other hand, persists for weeks to months and involves stable modifications such as DNA methylation, histone modifications, and small RNA activity. These epigenetic changes help plants maintain stress resistance without the need for continuous exposure to stress [59,60]. Transgenerational memory is transferred across generations where parental exposure to stress primes offspring, enhancing resilience. This inheritance is often mediated by epigenetic modifications, such as DNA methylation, that regulate stress-responsive genes [58].

A key consequence of plant memory is the modulation of secondary metabolite production. Epigenetic regulation has been shown to impact their biosynthetic pathways, altering metabolite concentrations and compositions in response to stress experiences [61]. For example, stress-induced DNA methylation changes can upregulate genes involved in flavonoid biosynthesis, enhancing antioxidant properties and plant immunity [62]. Additionally, plant hormones, such as jasmonic acid and salicylic acid, interact with epigenetic regulators to modulate secondary metabolite levels in response to biotic and abiotic stress [61].

### 5.2. Stress Recovery and Memory Retention

Plants possess remarkable capabilities to respond to environmental stress, recover, and retain a memory of these events. Balancing memory retention and resetting is critical for plant survival. Persistent memory can enhance stress resilience but may hinder recovery under favorable conditions. Mechanisms like RNA-directed DNA methylation (RdDM) facilitate this balancing [56,63].

Stress recovery involves the return of physiological processes to pre-stress conditions, whereas memory retention refers to the biochemical, molecular, and epigenetic “imprints” left behind by stress. These imprints allow plants to respond more rapidly and effectively when re-exposed to similar stress. For example, during drought, hypomethylation of promoter regions enhances the expression of phenylalanine ammonia-lyase (PAL), a key enzyme in secondary metabolite biosynthesis [64]. Once the stress subsides, these marks may remain, ensuring rapid gene reactivation upon subsequent stress exposure. Acetylation and methylation of histones regulate chromatin accessibility, modulating gene expression. Stress-induced histone acetylation (e.g., H3K9ac) leaves an “active” chromatin state near genes encoding secondary metabolites like flavonoids. This state can be preserved during recovery, enabling plants to maintain increased readiness for stress response [33]. miRNAs and siRNAs silence or activate specific stress-responsive pathways. Stress recovery often involves sustained expression of these small RNAs, which guide chromatin modifications to retain memory marks. For example, miR858 targets transcription factors involved in flavonoid biosynthesis, fine-tuning secondary metabolite production during and after stress [65].

Stress recovery is often marked by a “primed” metabolic state, where secondary metabolites such as flavonoids, terpenoids, and phenolics remain elevated. This metabolic memory contributes to quicker stress response upon re-exposure. For instance, anthocyanins, synthesized during UV stress, remain at higher baseline levels post-stress, enhancing photoprotection in subsequent UV exposure [66].

### 5.3. Regulatory Networks in Phenylpropanoid and Lignin Biosynthesis

The phenylpropanoid pathway is a main metabolic pathway that generates a diverse range of secondary metabolites, including flavonoids, lignins, stilbenes, and coumarins, all of which are essential for plant structural integrity and defense. Lignin biosynthesis, in particular, is tightly regulated and dynamically adjusted in response to developmental cues and environmental stress [67]. The regulatory networks controlling these pathways are multi-layered, involving a complex interplay between TFs, hormonal signaling pathways, and epigenetic mechanisms.

At the transcriptional level, R2R3-MYB transcription factors act as primary regulators of phenylpropanoid biosynthesis. For example, MYB46 and MYB83 are known “master switches” that activate the expression of cellulose, hemicellulose, and lignin biosynthesis genes by binding to secondary wall NAC binding elements [68,69]. These MYB factors themselves are regulated by upstream NAC transcription factors such as SND1 and NST1, which respond to developmental and stress-related signals [70]. WRKY transcription factors, particularly WRKY12 and WRKY13, modulate lignin biosynthesis by repressing or fine-tuning the activity of these master MYB regulators [38]. bHLH proteins, often acting as co-factors, enable control over pathway activation, ensuring specificity in response to different environmental stimuli [71].

Hormonal crosstalk significantly influences the regulatory balance of phenylpropanoid and lignin biosynthesis. Jasmonic acid and ethylene signaling pathways promote lignin biosynthesis under mechanical wounding and pathogen attack, while auxin and gibberellin signaling modulate the trade-off between growth and lignification [72]. Abscisic acid also plays a role in adjusting lignin accumulation during drought and salinity stress by activating ABA-responsive elements in the promoters of phenylpropanoid genes.

Epigenetic regulation of phenylpropanoid metabolism has gained increasing attention. Histone acetylation and methylation marks at the promoters of PAL, C4H, and 4CL genes influence the rate of pathway activation during stress [33]. In particular, H3K4me3 and H3K27me3 marks have been associated with dynamic activation and repression of lignin biosynthesis under changing environmental conditions [34]. Non-coding RNAs, such as miR858 and miR397, also modulate lignin pathway gene expression by targeting transcripts of MYB and laccase genes, respectively [39].

Post-translational regulation further refines pathway activity. Protein phosphorylation events, such as MAPK-mediated phosphorylation of MYB proteins, alter their stability and DNA-binding affinity, adding another regulatory dimension [73]. Together, these interconnected regulatory networks allow plants to fine-tune phenylpropanoid and lignin biosynthesis, balancing growth, defense, and energy allocation in response to biotic and abiotic stresses.

### 5.4. Transgenerational Memory and Metabolite Production

Transgenerational memory refers to the inheritance of stress-induced molecular and physiological changes by progeny (through several generations without additional exposure to stress) and highlights the evolutionary significance of plant memory in natural populations [55,74].

Research have [34] shown that flooding and drought in the parent environment lead to an increase in total aboveground biomass, reproductive biomass, height, and number of leaves in the offspring. Transgenerational effects of increased fitness were observed in offsprings of rye (*Secale sylvestre*) in cases where mother plants were treated with drought, which was translated into higher shoot biomass and seed yield in their offspring when exposed to drought [75]. Furthermore, compared to offspring that were not exposed to drought, plants whose parents experienced drought stress were more drought tolerant [76]. The transgenerational effects observed by [34] could also influence the production of secondary metabolites in offspring. When parent plants experience stressors like flooding or drought, they may upregulate the production of certain secondary metabolites (such as flavonoids, alkaloids, or terpenes) that help them cope with those conditions. These metabolites not only aid the stressed plants but may also be passed down to their offspring.

A whole-genome bisulfite sequencing analysis of heat-exposed and control offspring was used to investigate genetic variation (SNPs and INDELs) and epigenetic variation (DMPs and DMRs) of plants exposed to heat stress for 25 consecutive generations [77]. In stressed offspring, multigenerational heat stress produced phenotypic resilience. In particular, the parallel, unstressed control offspring did not perform as well as the stressed offspring. The fresh and dry weights of plants from the 25th generation were higher than those of control progeny following heat stress exposure. Heat stress and salinity stress can lead to the activation of specific metabolic pathways that enhance the production of secondary metabolites. The epigenetic changes, such as DNA methylation (DMPs and DMRs), observed in the stressed offspring (F2H, F25H) suggest that these modifications are linked to the regulation of genes involved in secondary metabolite biosynthesis. It has been observed that under high salinity stress, the direct progeny from generations G2–G5 of stressed plants exhibit higher germination and survival rates than those from generation G1 [78]. Additionally, phenotypic changes like leaf number and flowering time may be influenced by heat-induced changes in gene expression as well [79].

Along with the previously discussed abiotic stresses, biotic stresses like herbivory can induce heritable, adaptive phenotypic changes. Greater resistance to herbivores was shown by *Raphanus raphanistrum* seedlings from herbivore-damaged plants or those treated with jasmonic acid, characterized by increased trichome density and improved biochemical defenses [80,81]. When compared to genetically identical offspring of undamaged control plants, *Mimulus guttatus* plants with leaves experimentally harmed by herbivores exhibited a greater density of defensive leaf trichomes [82]. Trichomes play a crucial role in plant defense by producing and storing secondary metabolites, such as alkaloids, flavonoids, and terpenes, which help deter herbivores and pathogens. Increased trichome density in the offspring of *M. guttatus* mother plants exposed to simulated herbivory was linked to epigenetically inherited alterations in the transcription factor MYB expression [83]. Based on these observations, it is likely that the high trichome density trait is passed down through generations due to DNA methylation. For instance, MYB transcription factors are known to regulate the production of flavonoids and other phenolic compounds, which contribute to plant defense mechanisms. The transgenerational inheritance of increased trichome density may, therefore, also enhance the synthesis of these protective compounds, providing offspring with a pre-adapted defense response to herbivory [84].

Stress-induced methylation marks are often retained in germ cells, ensuring their transmission to progeny. For example, in *Arabidopsis thaliana*, salt stress leads to hypermethylation of transposable elements, stabilizing the genome and indirectly regulating stress-responsive genes in the next generation [56]. Repressive marks like H3K27me3, which silence stress-responsive genes during favorable conditions, are inherited through cell division and gametogenesis. These marks can be removed or modified during stress in progeny, enabling a rapid response to recurring stress [33]. Small RNAs, such as siRNAs, are packaged into germ cells and guide DNA methylation or histone modifications in the offspring. For instance, stress-induced siRNAs targeting transposons stabilize epigenetic landscapes, ensuring progeny resilience.

### 5.5. Stress-Induced Secondary Metabolite Synthetic Pathway Activation

Secondary metabolites, including phenolics, flavonoids, terpenoids, alkaloids, and phytoalexins, play crucial roles in mitigating stress by serving as antioxidants, osmoprotectants, and signaling molecules. Stress-induced activation of these pathways involves a complex interplay of signal perception, transcriptional regulation, and epigenetic modifications, ensuring a coordinated response tailored to specific environmental challenges. Abiotic stresses such as drought, salinity, and UV exposure trigger epigenetic changes that activate specific metabolic pathways. For example, methyl jasmonate-induced histone modifications have been linked to increased production of terpenoids [66,85].

Stress-induced pathway activation begins with the perception of environmental stimuli through membrane-bound receptors, such as PRRs or phytohormone receptors. Abiotic stresses like drought and salinity increase the production of ROS, triggering signaling cascades involving calcium influx and MAPKs. These early events activate TFs that regulate genes encoding secondary metabolite biosynthetic enzymes.

For example, drought stress increases the accumulation of ABA, a key phytohormone, which activates ABA-responsive transcription factors such as ABF and DREB. These TFs upregulate genes involved in flavonoid biosynthesis, leading to the production of quercetin and kaempferol, compounds that scavenge ROS and stabilize cellular membranes [66]. Similarly, UV radiation triggers the activation of UVR8, a UV-B photoreceptor, which initiates the synthesis of anthocyanins, a group of flavonoids with photoprotective properties.

Stress-responsive TFs play a pivotal role in orchestrating the activation of secondary metabolite pathways. Members of the MYB, bHLH, WRKY, and NAC transcription factor families are often recruited to regulate the expression of biosynthetic genes. These TFs bind to specific promoter regions of genes encoding enzymes such as chalcone synthase (CHS), phenylalanine ammonia-lyase (PAL), and terpene synthase (TPS), which are involved in the biosynthesis of flavonoids, phenolics, and terpenoids, respectively. For instance, MYB TFs interact with bHLH and WD40 proteins to form regulatory complexes that enhance anthocyanin production under UV stress. Similarly, WRKY TFs are induced during pathogen attack, activating phenylpropanoid pathways to produce lignin and phenolic acids, which reinforce cell walls and inhibit pathogen spread [66]. The coordinated activation of these pathways ensures rapid and robust production of secondary metabolites tailored to specific stress conditions.

DNA methylation is often altered in response to stress, either repressing or activating target genes. For example, hypomethylation of the PAL promoter region during drought stress enhances phenylpropanoid production, while hypermethylation of unrelated pathways conserves energy [64]. Histone acetylation, particularly at H3K9 and H3K27 residues, is associated with active chromatin states, facilitating the transcription of key biosynthetic genes. In *Camellia sinensis*, UV-induced histone acetylation at chalcone synthase genes promotes the synthesis of catechins, which provide photoprotection and antioxidant defense [86].

Small RNAs, including miRNAs and siRNAs, also contribute to stress-induced regulation. These molecules guide chromatin modifications and transcript degradation, ensuring precise control over secondary metabolite pathways. For instance, miR156 regulates SPL transcription factors, modulating flavonoid biosynthesis during recurrent heat stress [65].

Metabolomic memory includes the retention of the ability of stress-induced metabolic changes to persist beyond the initial stress event, allowing plants to respond more effectively to subsequent stresses. This memory can be classified into transient memory, which is short-lived and fades after the stress subsides, and transgenerational memory, which is inherited by subsequent generations. Both forms of memory are regulated by intricate epigenetic mechanisms, ensuring that metabolic pathways are primed for efficient and rapid activation under repetitive stress conditions.

Transient metabolomic memory plays a crucial role in the immediate aftermath of stress exposure, enabling plants to maintain a heightened state of metabolic activity for a limited duration. During stress, plants reprogram their metabolism to accumulate protective secondary metabolites such as flavonoids, phenolics, terpenoids, and alkaloids. These compounds serve as antioxidants, osmoprotectants, and signaling molecules, mitigating the effects of stress and preparing the plant for future challenges. For example, flavonoids accumulate during drought stress to scavenge ROS and stabilize cellular structures, and this elevated flavonoid content persists during recovery periods [66]. Similarly, phenolic acids like caffeic acid derivatives provide oxidative stress tolerance during and after exposure to drought [87]. Transient metabolomic memory ensures that these metabolites remain at elevated levels, allowing plants to respond more effectively to subsequent stress.

In contrast, transgenerational metabolomic memory extends beyond the lifespan of the stressed plant, equipping its progeny with enhanced resilience to similar stress conditions. Stress-induced epigenetic marks, such as DNA methylation and histone modifications, are stably inherited through gametes, creating a molecular framework for stress adaptation in subsequent generations. For example, drought-stressed *Medicago truncatula* plants transmit epigenetic modifications to their offspring, leading to elevated baseline levels of flavonoids and phenolic compounds that enhance their stress tolerance [88]. Similarly, stress-induced hypomethylation of regulatory regions in secondary metabolite biosynthetic genes, such as those involved in the jasmonic acid and salicylic acid pathways, have been observed in *Arabidopsis*, providing a molecular basis for inherited stress tolerance [56].

Histone modifications also play a significant role in transgenerational memory. Repressive marks such as H3K27me3 are maintained in progeny to conserve energy under non-stress conditions while priming stress-response genes for activation when needed. For example, in plants exposed to herbivory, the retention of H3K27me3 in jasmonate biosynthesis genes ensures controlled terpenoid production in progeny plants, enhancing their resistance to repeated herbivore attacks [33]. RNA-directed DNA methylation (RdDM) further stabilizes stress-responsive epigenetic landscapes by guiding small RNAs to target specific genomic regions, ensuring that stress-responsive pathways remain active across generations [87].

Plants exposed to repetitive stress exhibit a phenomenon known as priming, where they respond faster and more robustly to recurring stress compared to naïve plants. This enhanced response is mediated by the integration of transient and transgenerational memory mechanisms. In *Camellia sinensis*, for instance, UV and drought stress prime transcription factors involved in catechin biosynthesis, enabling rapid reactivation of these pathways upon re-exposure [86]. The interplay between DNA methylation, histone modifications, and non-coding RNAs creates a dynamic epigenetic landscape that adapts to stress conditions, ensuring optimal resource allocation and metabolic efficiency during repetitive stress events.

Secondary metabolite activation is further modulated by the integration of hormonal and environmental signals. Phytohormones such as JA, SA, and ET play synergistic or antagonistic roles in fine-tuning metabolite production. For example, JA and SA coordinate the production of phytoalexins like camalexin and flavonoids during pathogen attack, while ET modulates terpenoid biosynthesis in response to abiotic stresses [87]. This hormonal crosstalk ensures a balanced metabolic response, minimizing resource expenditure while maximizing stress tolerance. Stress-induced activation of secondary metabolite pathways can persist beyond the stress event, creating a transient memory that primes plants for subsequent exposures. In some cases, these metabolic changes are transmitted to progeny through epigenetic inheritance, creating a transgenerational memory (Table 1). For example, progeny of drought-stressed *Medicago truncatula* plants exhibit elevated flavonoid levels, enhancing their resilience to water deficit conditions [88].

### 5.6. Temporal Dynamics of Epigenetic Changes Under Stress

The epigenetic response of plants to environmental stress is a dynamic process that unfolds over multiple temporal scales. Immediately upon stress perception, rapid chromatin changes occur, including histone acetylation and phosphorylation, enabling swift activation of stress-responsive genes. For instance, within minutes to hours, increases in histone marks such as H3K9ac and H3K27ac at promoters facilitate transcription initiation, while phosphorylation of histone H2A contributes to chromatin relaxation [33]. Subsequently, small RNAs, including stress-induced siRNAs and miRNAs, are produced rapidly to mediate post-transcriptional gene silencing and fine-tune the expression of key metabolic genes [94].

In the short-term, over the course of hours to days, more stable modifications such as DNA methylation changes are established. These changes frequently occur at promoters of genes involved in secondary metabolism, such as phenylpropanoid and flavonoid biosynthetic genes, as well as at transposable elements. Such methylation changes ensure silencing of unnecessary genomic elements and optimize the allocation of resources toward stress response pathways [30,78]. Chromatin remodelers, including SWI/SNF complexes, are also recruited during this period, repositioning nucleosomes to sustain or repress transcription as needed [94].

Long-term responses, extending from weeks to months, involve the establishment of more persistent epigenetic marks that confer stress memory. Histone methylation patterns, including the sustained presence of H3K4me3 or reduction in repressive marks like H3K27me3, have been associated with the long-term preparedness of plants for recurring stress [37]. Additionally, DNA methylation alterations can influence developmental processes such as flowering time, root growth, and metabolic reprogramming, ensuring the plant is better equipped for anticipated future challenges [32].

Some epigenetic changes are not limited to a single generation. Studies in *Arabidopsis* and rice have demonstrated that the offspring of stressed plants exhibit altered gene expression and increased resilience to drought and salinity, often mediated by the persistence of siRNAs that direct epigenetic reprogramming in germline cells [95,96].

Despite this persistence, the reversibility of epigenetic marks is an important feature of the regulatory system. Histone modifications are often transient, with HDACs and demethylases restoring chromatin states once the stress subsides [97]. However, stress memory genes tend to retain a controlled chromatin configuration, characterized by bivalent marks that balance repression and activation, enabling a more rapid transcriptional response to future stress episodes [98].

Interestingly, these temporal dynamics are integrated with the plant’s metabolic adjustments. Changes in chromatin structure at key metabolic genes coincide with shifts in carbon and nitrogen allocation toward the synthesis of protective secondary metabolites [29]. Moreover, signals from organelles, such as chloroplasts and mitochondria, influence chromatin remodeling activity, ensuring that the energy status and metabolic state of the plant are closely aligned with epigenetic regulation [46]. The fine-tuned orchestration of these immediate, intermediate, and long-term epigenetic responses underscores the plant’s ability to adapt to fluctuating environments while retaining memory of past experiences, an ability that holds great promise for improving crop resilience through epigenetic breeding strategies.

## 6. Applications and Future Directions

### 6.1. Biotechnological Implications

Secondary metabolites are naturally occurring, biologically active compounds produced by plants, essential for their growth, defense, and adaptation to environmental stresses. These stresses include rising temperatures, altered precipitation patterns, and more frequent extreme weather events driven by climate change, as well as exposure to harmful chemicals and pollutants resulting from human activities. Plant SMs are commonly utilized across various industries, such as agriculture, nutrition, cosmetics, and pharmaceuticals, due to their diverse range of beneficial properties [99,100]. SMs are particularly valuable for human health and well-being, exhibiting an impressive array of biological activities such as antibacterial, antioxidant, antifungal, anti-inflammatory, and anticancer effects [101]. Their multifunctional roles have made them essential components in the development of therapeutic agents and natural remedies. Recently, researchers have gained interest in SMs due to advancements in cutting-edge technology including various chromatography and high-resolution spectroscopy tools and MICS platforms [102]. Many of these compounds hold significant potential for developing new pharmaceuticals, contributing to the ongoing exploration of plant-based solutions for modern healthcare challenges. However, to meet the growing demand for therapeutically valuable natural compounds, researchers are exploring various advanced techniques to enhance the synthesis of secondary metabolites. These methods include callus culture, genetic engineering, hairy root culture, shoot culture, suspension culture, and elicitation [103]. Additionally, continuous development of novel technologies is the primary concern, as well as their integration to achieve optimal results. Through deeper understanding and application of these strategies, researchers aim to increase both the quantity and diversity of secondary metabolites, thereby expanding their potential applications across a wide array of industries and medical fields. Biotechnological tools can thus contribute to (a) enhanced yield and consistent production of valuable metabolites or whole crops; for example, (b) enhanced stress resistance and adaptability of plants, which is important for large-scale production systems where environmental control might be costly or challenging, (c) ensuring tailored metabolite profiles where the induction of specific epigenetic changes leads to the synthesis of specialized metabolites, (d) improving scientists’ understanding of complex metabolic networks, and (e) contributing to sustainable and green approaches in different industries.

### 6.2. Crop Improvement

Traditional crop breeding presents several challenges in the face of a rapidly changing climate and growing demands. These include long timeframes for developing new varieties, imprecise genetic modifications, difficulties in crossing genetically distant plants, and the frequent reliance on harmful chemical inputs such as fertilizers, pesticides, and herbicides, which can negatively impact the environment. Additionally, traditional breeding often struggles to produce crops with adequate resilience to biotic and abiotic stresses. Overcoming traditional approaches in agriculture and crop breeding in favor of epigenetics and modern biotechnology tools is therefore of paramount importance.

Epigenetic techniques are increasingly contributing to improved crop yields and resilience against biotic and abiotic stresses and anthropogenic pollutants. Manipulating miRNA pathways can enhance plant immunity, offering crop protection [104]. Plant SMs present viable alternatives to synthetic agrochemicals, playing crucial roles in plant defense. Volatile SMs attract pollinators while also supporting direct and indirect plant defenses [105]. Insecticidal SMs like pyrethrins and triterpene azadirachtin effectively deter insects with minimal toxicity to non-target organisms. Pyrethroids, derived from natural compounds, are key to synthetic pesticide development due to their efficiency, low residues, and safety for mammals [106]. As essential components of sustainable agriculture, SMs integrate seamlessly into integrated pest management. This is because SMs enhance plant defense by increasing the enzyme production and gene expression involved in resistance, with biosynthesis regulated by early defense signaling [2].

Plants’ phenotypic plasticity is driven by changes in gene expression, which are regulated by epigenetic modifications. These changes play a crucial role in how plants respond to stress and adapt to varying environments. Environmental factors like temperature, light, and water availability can trigger epigenetic changes in plants. By exposing plants to specific stress conditions, we can induce modifications in their epigenomes, leading to visible changes in traits. For example, DNA hypomethylation was observed in *Zea mays* [107] and *Brassica napus* under heat stress [108], while cold stress led to DNA hypermethylation in *Solanum lycopersicum* [51]. These examples show that plant responses to abiotic stress are not uniform; different species or even different regions within the same plant may exhibit varying methylation patterns.

Under biotic stress, pathogens can also reshape the epigenome. For instance, exposure to β-aminobutyric acid in *Arabidopsis* led to changes in histone acetylation and methylation, affecting defense gene expression [109]. Similarly, *Pseudomonas syringae* infection altered histone modifications, increasing defense gene expression [110]. Additionally, altered DNA methylation has been shown to play a key role in defense against *Hyaloperonospora arabidopsidis* [111].

The impact of heavy metals on crop quality has gained significant attention due to their negative effects on plant growth, photosynthesis, and overall health [112]. Plants can undergo dynamic epigenetic changes in response to environmental signals, and understanding and controlling these changes in real-time can help tailor plant responses to stresses, enhancing adaptive plasticity [113]. However, developing effective methods for delivering epigenetic modifiers to plants is key for practical applications.

There are many examples of how plants enhance tolerance to metals like Al, Cr, Cd, Cu, and As through epigenetic changes, showcasing the potential of these modifications in improving stress resilience. Changes in DNA methylation patterns can activate metal detoxification genes, boosting tolerance to metal stress [114]. Epigenetic modifications of stress-responsive promoters using demethylating agents also enhance metal transport and sequestration of gene activation. Histone methylation adjustments further improve the regulation of these genes, contributing to better cadmium tolerance. Additionally, siRNA-mediated RNA-directed DNA methylation has been shown to alter chromatin states and enhance chromium tolerance in wheat [115].

CRISPR/Cas-based epigenome editing has been applied to modify epigenetic markers linked to aluminum stress in soybeans, improving metal tolerance. Similarly, multiplex CRISPR strategies have been used to edit multiple epigenetic markers in sunflowers under combined heavy metal stress, demonstrating the potential for boosting overall plant resilience [116,117].

Epigenetic techniques hold great promise for the future of crop improvement, offering innovative solutions to enhance stress tolerance, improve resilience, and optimize agricultural productivity in an ever-changing environment.

### 6.3. Emerging Technologies

To understand the influence of epigenetics under simultaneous biotic and abiotic stresses, the complex interactions between plants and their environment, and to advance the progress in different industries, a wide range of epigenetic methods are being used. These include well-established methods that are still evolving, such as metabolic engineering, elicitation, cell cultures, in vitro mutagenesis, and graft-mediated reshaping to emerging techniques, which is the focus of this review (Table 2). These tools can elucidate the complex regulatory networks underlying secondary metabolism [56,63].

**Figure 3:**
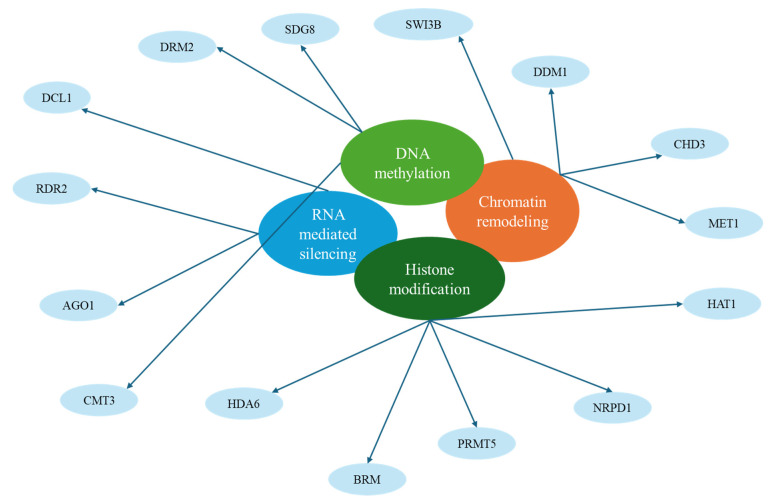
Key genes involved in plant epigenetic regulation of metabolite production.

The genes that play critical roles in mediating epigenetic modifications in order to ensure tolerance to pathogens in plants include HAC1, SDG8, MET1 and DRM2, AGO1 and DCL1, as well as SWI3B [128]. Some of these genes have synergistic roles, such as MET1 and DRM2 (Figure 3). Taken together, these genes highlight the adaptability of plant immunity through epigenetic regulation, allowing for dynamic responses to evolving pathogens [129,130].

MET1, CMT3, DRM2, and DDM1, regulate DNA methylation and maintain genomic stability under stress. HAT1, HDA6, SDG8, and PRMT5 are responsible for histone acetylation and methylation, altering chromatin structure to activate or repress gene expression. AGO1, DCL1, RDR2, and NRPD1 participate in RNA-mediated silencing pathways, guiding small RNAs to target stress-responsive genes. Chromatin remodeling is facilitated by SWI3B, CHD3, BRM, and DDM1, allowing plants to rapidly adjust transcriptional programs and secondary metabolite synthesis in response to environmental cues.

### 6.4. Opportunities and Challenges for Application

The targeted manipulation of SM biosynthesis genes holds significant potential not only for improving plant productivity and stress resilience but also for benefiting multiple industries such as pharmaceuticals, cosmetics, and food. Plants naturally boost enzyme production and gene expression of defense pathways in response to stress signals, a process that is tightly regulated by early perception mechanisms and epigenetic modifications. Leveraging these regulatory networks could enhance insect and pathogen resistance in crops, reduce reliance on chemical pesticides, and increase the yield of valuable bioactive compounds. In the pharmaceutical industry, stress-induced metabolites can serve as leads for novel drug development, offering antioxidant, anti-inflammatory, and anticancer properties. In cosmetics, phenolic compounds and terpenoids have applications as natural skin-protective agents. The food industry can utilize SMs as natural preservatives and flavor enhancers, contributing to cleaner-label products and consumer health benefits.

Biotechnological advances, including metabolic pathway engineering and the use of elicitors, enable the enhanced production of these metabolites in controlled conditions. However, challenges persist. In pharmaceutical research, one major obstacle is obtaining sufficient quantities of bioactive compounds for in vitro and in vivo testing, particularly from endangered or protected plant species. While cultivation of medicinal plants or using heterologous expression systems provides alternatives, these approaches sometimes fail to replicate the exact metabolic profiles observed in native environments [7]. Interestingly, plants exposed to climate stressors often show enriched phytochemical diversity, making them potential novel sources for drug discovery. For instance, under salinity stress, *Capsicum annuum* increases hydroxycinnamic acids, capsaicinoids, and flavonoids, which contribute to enhanced stress tolerance and defense [131].

Access to natural products is also constrained by legal frameworks and indigenous knowledge protection. Regulatory limitations and lack of standardized quality control hinder the industrial application of plant-based metabolites. Furthermore, the need to enhance crop yield and quality while minimizing environmental impact remains a persistent challenge, compounded by issues related to botanical resource sustainability, ecological conservation, and harmonization of regulatory policies [102].

From an epigenetic perspective, opportunities arise through the modulation of DNA methylation, histone modifications, and ncRNA pathways to influence SM biosynthesis and plant adaptability. However, improving the specificity of epigenetic editing remains a key technological hurdle. While CRISPR/dCas9-based epigenome editing has shown promise in modulating gene expression without altering DNA sequences, efficient delivery methods and minimization of off-target effects remain limiting factors [10]. The development of tissue-specific delivery systems using nanocarriers or viral vectors could overcome these barriers. Furthermore, the lack of complete epigenetic maps for many crop species restricts precision-targeting strategies. The integration of epigenomics with transcriptomics and proteomics can provide a more comprehensive understanding of stress responses and help to identify master regulators for targeted interventions [8].

Field validation of epigenetically modified plants is still in its infancy, and the long-term stability of introduced epigenetic marks under natural environmental conditions is not well understood. Additionally, ethical and ecological concerns must be carefully considered. There is a risk that unintended epigenetic modifications could alter plant–microbe interactions, disrupt local ecosystems, or contribute to invasiveness. Moreover, the potential for these changes to propagate through wild populations raises questions about the long-term societal and environmental consequences [37].

Exploring epigenetic variation triggered by both abiotic and biotic stresses offers a powerful avenue for precision breeding and climate-smart crop development. By harnessing stress-induced epigenetic plasticity, it may be possible to improve stress tolerance and productivity without introducing foreign DNA, thus offering an alternative to conventional GM approaches. However, techniques like whole-genome bisulfite sequencing and ChIP-seq are resource-intensive, particularly for species with large, complex genomes. The future will rely on advances in high-throughput technologies, computational modeling, and field-based multi-omics studies to optimize these approaches and translate laboratory findings into robust, sustainable agricultural solutions.

Despite substantial progress, current research on epigenetic regulation of secondary metabolite biosynthesis under stress displays notable gaps and methodological limitations. Many studies, particularly those conducted on model plants like Arabidopsis thaliana, provide important mechanistic insights, yet their transferability to crop species remains uncertain due to significant genomic and epigenomic complexity. Moreover, while studies have demonstrated the potential of CRISPR/dCas9-based epigenome editing, challenges with delivery specificity, off-target effects, and stable inheritance of induced modifications limit current applications in large-scale breeding programs.

Another critical limitation is the scarcity of high-resolution, time-resolved epigenomic studies that map histone modifications and DNA methylation dynamics throughout different stress phases. Furthermore, while research has highlighted the involvement of small RNAs in epigenetic inheritance [39], there is limited evidence linking specific sRNAs to consistent alterations in secondary metabolite profiles in field conditions.

The available literature also lacks comparative studies evaluating the ecological consequences of stress-induced epigenetic changes. While increased metabolite production can enhance crop resilience, potential trade-offs in growth, reproductive success, and interactions with beneficial microorganisms are not thoroughly investigated. Therefore, future research must prioritize crop-specific epigenetic studies, integrate multi-omics data, and conduct long-term field trials to validate laboratory findings.

## 7. Conclusions

Epigenetic regulation plays a crucial role in modulating secondary metabolite biosynthesis and enhancing plant resilience to environmental stresses. Through DNA methylation, histone modifications, and small RNA pathways, plants establish stress memory, enabling rapid and efficient responses to recurring challenges. This molecular memory can be transgenerational, influencing metabolite production and stress tolerance in offspring, with significant implications for crop improvement and sustainable agriculture.

The ability to epigenetically prime plants for stress presents a promising strategy for reducing reliance on chemical inputs while enhancing plant defense mechanisms. Advances in epigenome editing and multi-omics approaches offer new opportunities to manipulate secondary metabolite pathways for applications in pharmaceuticals, nutraceuticals, and biotechnology.

Future research should focus on deciphering the precise regulatory networks of epigenetic modifications and developing innovative tools to harness these mechanisms for improving crop yield, phytochemical production, and climate resilience. By leveraging plant epigenetics, we can enhance agricultural sustainability and food security, making crops more adaptable to changing environmental conditions while optimizing their biochemical potential.

## Figures and Tables

**Table 1:** Examples of plants with stress memory regulating secondary metabolite synthesis in progeny.

Plant Species	Stress Type	Secondary Metabolite	Identified Epigenetic Control	Memory in Progeny?
*Artemisia annua* [89]	Water deficit	Artemisinin	DNA methylation and histone acetylation	No clear evidence
*Sorbus pohuashanensis* [90]	Biotic stress (yeast extract)	Biphenyl phytoalexins	Altered histone marks and DNA methylation	Yes, short-term
Mosses (*Pogonatum cirratum*, *Hypnum plumaeforme*) [91]	Low-temperature stress	Phenylpropanoids, triterpenes	DNA methylation	Partial, species-specific
Lamiaceae (*Rosmarinus officinalis*, *Salvia officinalis*, etc.) [92]	Recurrent drought stress	Essential oils (α-pinene, D-limonene, eucalyptol)	Histone methylation	Yes, short-term
*Camellia sinensis* [86]	UV and drought stress	Catechins, L-theanine	Histone acetylation and DNA methylation	Not clear
*Catharanthus roseus* [87]	Drought stress	Vinblastine, vincristine	Histone methylation and chromatin remodeling	Yes, transgenerational
*Medicago truncatula* [88]	Salinity stress	Flavonoids	Chromatin remodeling and miRNA regulation	No
*Thymus vulgaris* [92]	Reduced irrigation	Thymol, carvacrol	Epigenetic priming (DNA methylation)	Yes, short-term
*Glycine max* (soybean) [93]	Drought stress	Isoflavonoids	DNA methylation and histone modifications	Yes, transgenerational
*Populus tremula* [87]	Pathogen attack	Salicylates	DNA demethylation	Yes, transgenerational
*Nicotiana tabacum* [65]	Drought and salinity stress	Alkaloids (e.g., nicotine)	Small RNAs and chromatin remodeling	Not clear

**Table 2:** Overview of emerging epigenetic methods and possible applications.

Method	Description	Application Examples
miRNAs [104,117]	MicroRNAs (miRNAs), small non-coding RNAs, regulate gene expression at the post-transcriptional level and are key players in plant–pathogen interactions. They control immune responses, stress signaling, and cross-kingdom communication. miRNAs interact with other epigenetic mechanisms, like DNA methylation and histone modifications, influencing chromatin dynamics and gene regulation. Environmental factors, such as temperature and drought, can also impact miRNA activity during pathogen attacks. Understanding and manipulating miRNA pathways offers potential for improving plant immunity and developing new crop protection strategies.	mi393—One of the most studied miRNAs in plant immunity. mi398—Involved in regulating oxidative stress during pathogen attack.
Exo-RNAi [118,119,120]	RNAi has gained attention in agriculture for enhancing disease resistance, plant development, and crop traits. Traditionally, this was achieved by introducing transgenes into plants, but resistance to genetically modified (GM) crops has limited their use. As a non-transgenic alternative, researchers now use exogenous RNA molecules (exo-RNAi) in plants. Exogenous dsRNAs, siRNAs, or hpRNAs can activate RNAi and protect plants from pathogens such as viruses, fungi, and insects. RNAi is initiated when dsRNA is processed into small interfering RNAs (siRNAs) by dicer-like endonucleases. Methods for applying RNA molecules include spraying, injection, infiltration, and soaking. The main limitation of exogenously applied naked dsRNAs is their short stability.	External application of dsRNAs and siRNAs has proven effective in protecting plants like barley, tomatoes, strawberries, and soybeans from fungal pathogens.Co-injecting dsRNAs targeting the replicase protein gene of *Pepper mild mottle virus*, *Tobacco etch virus*, and *Alfalfa mosaic virus* into tobacco leaves significantly reduced viral infections.RNAi-based genetic transformation targeting the carotenoid cleavage dioxygenase gene aimed to regulate branch growth and increase the number of branches in kiwi plants.
epiRILs [118,121,122]	epiRILs are crucial for crop improvement and genetics, helping researchers to understand epigenetic effects like DNA methylation on plant traits. They are valuable for studying the heritability of epigenetic traits and exploring the use of epigenetic variation in crop breeding to advance improvement strategies.	Studies with epiRILs in *Arabidopsis* have shown that stress-induced epigenetic changes can be heritable, providing phenotypic plasticity that helps plants withstand stress.
CRISPR/Cas [93]	This gene-editing technology is still relatively new but rapidly advancing and revolutionizing the ability to modify plant genomes with precision, especially for secondary metabolite production.	CRISPR/Cas-based epigenome editing improved salt tolerance in maize by modifying epigenetic markers, altering gene expression for ion homeostasis and osmotic control. In wheat, epigenetic changes under heat stress induced transgenerational stress memory, enhancing long-term heat tolerance.
Inhibition of DNA methylation [118,123,124,125]	This method targets DNA methylation to regulate gene expression and plant traits, including secondary metabolite pathways. Compounds like 5-AzaC and zebularine, non-methylable cytidine analogs, are commonly used, though researchers are exploring more stable alternatives.	In addition to *Arabidopsis*, many plant species have been studied using 5-aza or aza-dC to explore DNA methylation’s role. In carrots, 5-aza suppressed embryogenic cell clump formation. It induced flowering in *Silene armeria*, *Pharbitis nil*, and *Perilla frutescens*, independent of photoperiodic conditions. In *Solanum ruiz-lealii*, 5-aza induced early flowering and leaf morphology changes, likely due to increased miR172 transcription.
Inhibition of histone deacetylation and demethylation [49,118,126,127]	The second major group of histone deacetylase inhibitors includes amino-benzamides, hydroxamic acids, short-chain fatty acids, and cyclic peptides. Compounds like butyrate and trichostatin A inhibit histone deacetylation. Trichostatin A and suberoylanilide hydroxamic acid inhibit HDAC, increasing histone acetylation and loosening chromatin to activate stress-responsive genes. Small molecules targeting histone acetyltransferases can also enhance acetylation, promoting an open chromatin state for stress-related gene expression.	These epigenetically active drugs are now primarily used in plant tissue culture therapy.Histone acetylation changes in drought-stressed rice enhanced drought-related gene expression, improving water-use efficiency and resistance.

## Data Availability

No new data were created or analyzed in this study. Data sharing is not applicable to this article.
